# Hidden Allee effect in photosynthetic organisms

**DOI:** 10.1080/19420889.2020.1800999

**Published:** 2020-08-30

**Authors:** Hiroshi Ohkawa, Chiharu Takatsuka, Tomonori Kawano

**Affiliations:** a Faculty of Agriculture and Life Science, Hirosaki University, Hirosaki, Japan; b International Photosynthesis Industrialization Research Center, Faculty of Environmental Engineering, The University of Kitakyushu, Kitakyushu, Japan; c Advanced Photonics Technology Development Group, RIKEN Center for Advanced Photonics, Saitama, Japan

**Keywords:** Allee effect, cyanobacteria, green paramecia, logistic equation, photosynthesis

## Abstract

In ecology and population biology, logistic equation is widely applied for simulating the population of organisms. By combining the logistic model with the low-density effect called Allee effect, several variations of mathematical expressions have been proposed. The upper half of the work was dedicated to establish a novel equation for highly flexible density effect model with Allee threshold. Allee effect has been rarely observed in microorganisms with asexual reproduction despite of theoretical studies. According to the exploitation ecosystem hypotheses, plants are believed to be insensitive to Allee effect. Taken together, knowledge on the existence of low-density effect in photosynthetic microorganisms is required for redefining the ecological theories emphasizing the photosynthetic organisms as the basis for food chains. Therefore, in the lower half of the present article, we report on the possible Allee effect in photo-autotrophic organisms, namely, green paramecia, and cyanobacteria. Optically monitored growth of green paramecia was shown to be regulated by Allee-like weak low-density effect under photo-autotrophic and photo-heterotrophic conditions. Insensitiveness of wild type cyanobacteria (*Synechocystis* sp. Strain PCC6803) to low-density effect was confirmed, as consistent with our empirical knowledge. In contrast, a mutant line of PCC6803 impaired with a photosynthesis-related *pxcA* gene was shown to be sensitive to typical Allee’s low-density effect (*i.e*. this line of cells failed to propagate at low cellular density while cells start logarithmic growth at relatively higher inoculating density). This is the first observation that single-gene mutation in an autotrophic organism alters the sensitivity to Allee effect.

## Introduction

In biological systems, several types of density effects are known. Density of self and non-self (as biomass or individuals) may affect or alter the propagation rate, morphological developments [such as fungal spore germination control]; [1], and metabolic regulations [as in bacterial and fungal quorum sensing events]; [2, 3] of living organisms throughout the lifecycle through competition and/or inter-cooperation.

One of the most recognized modes of density effects is Allee effect which was named after the works of an ecologist, W. C. Allee [4], etc.]. The upper half of the present perspective work was dedicated to establish a novel equation for highly flexible low-density effect model with Allee threshold (*A*).

In the lower half of the present study, we aimed to analyze the growth of two photosynthetic organisms, namely green paramecia (*Paramecium bursaria*) and cyanobacteria (*Synechocystis* spp., strain PCC6803) using a newly proposed logistic equation-derived mathematical model designed for quantifyig the extent of possible growth regulation due to density effect, since knowledge on the existence of Allee-like low-density effect in photosynthetic microorganisms is required for redefining the exploitation ecosystem hypotheses in which the population dynamics of photosynthetic organisms is emphasized as the basis for the discussion on the length of food chains as discussed in the later sections.

## Bases of discussion on the logistic models reflecting Allee effect

### Conventional logistic models

Today, logistic kinetics are widely applied to the various cases in interdisciplinary fields, chiefly statistics, demography, ecology, and bio-mathematics related to population dynamics [5]. Historically, microbiologists [6] and demographists [7] observed that the logistic equation could be commonly applied to simulate the growth of microorganisms and human population. By defining the rate of intrinsic increase (*r*) and carrying capacity (*K*) as key factors determining the changes in population size (*N*), following well-known form of logistic model (1) was formulated and refined by Lotka [8] and Volterra [9].

(1) $$\frac{dN}{dt}=rN\left(\frac{K-N}{K}\right)$$

When an organism of interest obeys the conventional logistic model expressed as equation (1), the organism keeps growing until attaining the upper limit density defined as *K*. In addition to the upper limit of population growth (*K*), we can assume the lower threshold of population density, nowadays known as Allee threshold (*A* or *A* value), above which population growth is allowed and accelerated as population density increases until interfered by the upper limit effect by *K*, and below which the growth ceases down and the population gradually approaches to zero. Such low-density effect is known as Allee effect. As discussed above, two distinct types of population density effects are observed around *K* and *A*, thus, mathematical formulations combining the effects of *K* and *A* are required.

There are several variations in mathematical expressions derived for describing the different modes of Allee effects [10,11]. A mode of low-density effect often referred to as ‘strong’ Allee effect can be expressed by conjugating a term for low-density effect, (*N* − *A*)/*A*, with the conventional logistic equation as below [12]:

(2) $$\frac{dN}{dt}=rN\left(\frac{K-N}{K}\right)\left(\frac{N-A}{A}\right)$$

where *A* is the critical lower limit of population size known as Allee threshold. The lesson we can learn from this model is that, at ecological scale, organisms share the macroscopic resilience upon facing the transiently forced declines of population, possibly regaining its population size within the range between *A* and *K*.

On the other hand, the mode of low-density effect known as ‘weak’ Allee effect can be expressed by using a distinct term (*N* − *A*)/*K*, thus, equation can be rearranged as below.

(3) $$\frac{dN}{dt}=rN\left(\frac{K-N}{K}\right)\left(\frac{N-A}{K}\right)$$

### Theoretical background for Allee models with flexibility

Due to the increased flexibility in the choice for the size of *A* value ranging between 0 and *K* (or even below 0), the ‘weak’ Allee model expressed with equation (3) is also referred to as ‘flexible’ Allee equation. By introducing *A* = 0, the above equation (3) can be modified into equation (4), as previously discussed elsewhere [13].

(4) $$\frac{dN}{dt}=\frac{rN^{2}}{K}\left(1-\frac{N}{K}\right)$$

It is trivial that this equation can be further simplified into the following form.

(5) $$\frac{dN}{dt}=\frac{rN^{2}}{K^{2}}\left(K-N\right)$$

In fact, these specific equations (Eqs. 4 and 5) allow very interesting modes of the population dynamics, namely, apparent ‘Allee effect without Allee threshold’ can be expressed, through which the positive density effect (growth acceleration) occurs as the population approaches *K* (by countracting the suppression by high-density effect due to the upper size limit defined by *K*), and negative density effect (growth suppression) becomes dominant as the population size stays close to zero.

To confer higher generality to this mathematical model, the exponent 2 used in equation (5) can be replaced with any given exponent *α* (either integer or non-integer) as below.

(6) $$\frac{dN}{dt}=\frac{rN^{\alpha }}{K^{\alpha }}\left(K-N\right)$$

Again, the newly arranged equation (6) allows expressing the Allee-type of density effects without defining Allee threshold. It is noteworthy that this novel model allows the use of exponent, not only 2, but also any number, even below 1. When the exponent is 1, the equation is identical to the conventional logistic equation (1). By setting an exponent within the range between 1 and 2, weaker extent of the Allee’s density effect can be expressed. In contrast, by introducing an exponent greater than 2, relatively stronger extent of the Allee’s density effect can be expressed. Interestingly, exponent *α* in equation (6) can be shrunk down even to below 1, resulting in weakening of the intrinsic low-density effect attributed to the classical logistic model without Allee effect. While the extent of density effect is likely fixed in the conventional Allee models (Eqs. 2 and 3), the novel Allee equation (6) allows expression of a broader range of density effects. Note that this novel equation can be used to express both the obverse and reverse sides of Allee effects without assuming the presence of Allee threshold.

### Reintroduction of Allee threshold to the flexible density model

As above, we consider the usefulness of the flexible density effect model as expressed by equation (6). In order to confer further generality to the model, it is tempting to reintroduce the room for *A* value (Allee threshold) in the flexible density effect model. By conjugating equation (6) with the term for Allee threshold, (*N* − *A*)/*K*, novel equation for describing the flexible density effect with defined Allee threshold can be designed as below.

(7) $$\frac{dN}{dt}=\frac{rN^{\alpha }}{K^{\alpha +1}}\left(K-N\right)\left(N-A\right)$$

## Allee effect in photo-autotropic model organisms

### Allee effect in plants and microorganisms?

Although the presence of *K* values has been observed in a variety of organisms ranging from unicellular microbes to multicellular higher organisms, the presence of *A* value has been rarely observed in microorganisms. While the organisms with sexual propagation requiring mating partners are apparently sensitive to Allee-type of low-density effect [14], theoretical studies have pointed out that Allee effect can be found even among microbial communities in which propagation proceeds through cell division basically without mating process [15]. However, the only experimental demonstration reported today, in support of Allee effect functioning in bacterial species, was the case from genetically engineered model in *E. coli* [16]. Similarly, among the protozoan unicellular microorganisms, a case in *Tetrahymena* was the only case demonstrating the Allee effect [17].

According to the exploitation ecosystems hypotheses, the population sizes of photosynthetic organisms are the bases for determining the length of food chains [18,19]. However, among higher organisms, plants are believed to be insensitive to Allee-type of low-density effects, since Rosenzweig [20], who studied the population dynamics of three species in the three-trophic-level exploitation system (consisted of plants, herbivores, and carnivores), showed assumption that plants are not directly harmed by low density, meaning that there is no true Allee effect in plant community.

### Possible density effect tested in green paramecia and cyanobacteria

In the present perspective article, we wish to discuss if the growth of microbial photosynthetic organisms is affected by Allee-type of density effects or not, based on the preliminary dataset obtained from two distinct model organisms, namely, green paramecia (*Paramecium bursaria*) and cyanobacteria (*Synechocystis* spp. PCC6803). We have previously established the model for evolutionary emergence of photosynthetic organisms through development of endosymbiotic relationship between protozoan cells and photosynthetic bacteria, by employing apo-symbiotic green paramecium cells and *Synechocystis* spp. PCC6803 as a pair of model organisms for forced symbiosis mimicking the evolutionary event where phagocytic intake of PCC6803 cells into *Paramecium* cell body followed by intracellular propagation of PCC6803 took place under the laboratory conditions [21].

*Paramecium* species including green paramecia have been used for testing the usefulness of logistic model as Gause [22] documented the classical works focusing on the competition for survival among several *Paramecium* species. Among *Paramecium* species, green paramecia is the only photosynthetic species harboring the cells of *Chlorella*-like endo-symbiotic green algae [23]. In fact, like most of unicellular microorganism, a single cell of green paramecia in an isolated environment possess potency for cell division and propagation as *Paramecium* strains are often isolated from environmental freshwater samples through manual single cell-based isolation and propagation in hanging drops of medium [24]. Therefore, there should be no absolute low-density limit for this organism. Therefore, we expected relatively loose density effect other than strict Allee-type density effect often found in higher animals.

Cyanobacterial strains would be the best biological models for studying the origin of photosynthetic organisms leading to evolution of green plants, since the photosynthetic apparatus found in cyanobacteria is structurally and functionally similar to chloroplasts in terrestrial plants [25]. Among cyanobacteria, *Synechocystis* sp. PCC6803 is one of the most highly studied models capable of growth under both autotrophic and heterotrophic conditions [26].

The use of mutants of transformable strains is a powerful technique for studying the photosynthetic mechanism in cyanobacteria such as CO_2_-concentrating mechanism [27]. The *pxcA* gene (formerly known as *cotA*, homolog of *cemA*) isolated from *Synechocystis* PCC6803 is coding for a chloroplast envelope membrane-localized protein, localized in chloroplast genome.  Reportedly, *PxcA* mutant impaired with *pxcA* gene (hereafter, referred to as Δ*pxcA*), is not capable of proton extrusion upon exposure to light and CO_2_ transport [27,28]. According to earlier works [27, etc], *pxcA* gene and its homologues are believed to be acquired in the course of evolution by cyanobacteria. Therefore, comparison between wild type and Δ*pxcA* mutant may reveal the key role of *pxcA* gene in evolution of photosynthesis-dependent growth of cyanobacteria.

### Growth kinetics of green paramecia with and without food bacterium

The cells of green paramecia (strain, KN-21) were propagated under *ca*. 3500 lux of natural-white fluorescent light through 12 h:12 h of light-dark cycle at 23ºC, with and without supplementation of food bacteria as described elsewhere [24], with minor modifications as below. Briefly, bacterized medium was prepared by inoculating the yeast extract tablet-based medium (EBIOS medium, 1 tablet/L, autoclaved) with noninfectious strain of *Klebsiella pneumoniae* (food bacteria) 1 day prior to the addition to the ciliate culture. On a sheet of nylon mesh (pore size, 10 μm), the cells of green paramecia were collected from the large-sized pre-cultures, then, washed and resuspended in fresh EBIOS medium at various cellular densities ranging from 1 to 1000 cells/ml in fresh EBIOS medium.

For the cultures with food bacterium, the cellular suspension of green paramecia and 1 day-old bacterized medium were mixed at 1:1 mixing ratio, while bacteria-free cultures were prepared by dilution of green paramecia with aliquot of fresh EBIOS medium.

**Figure 1.**
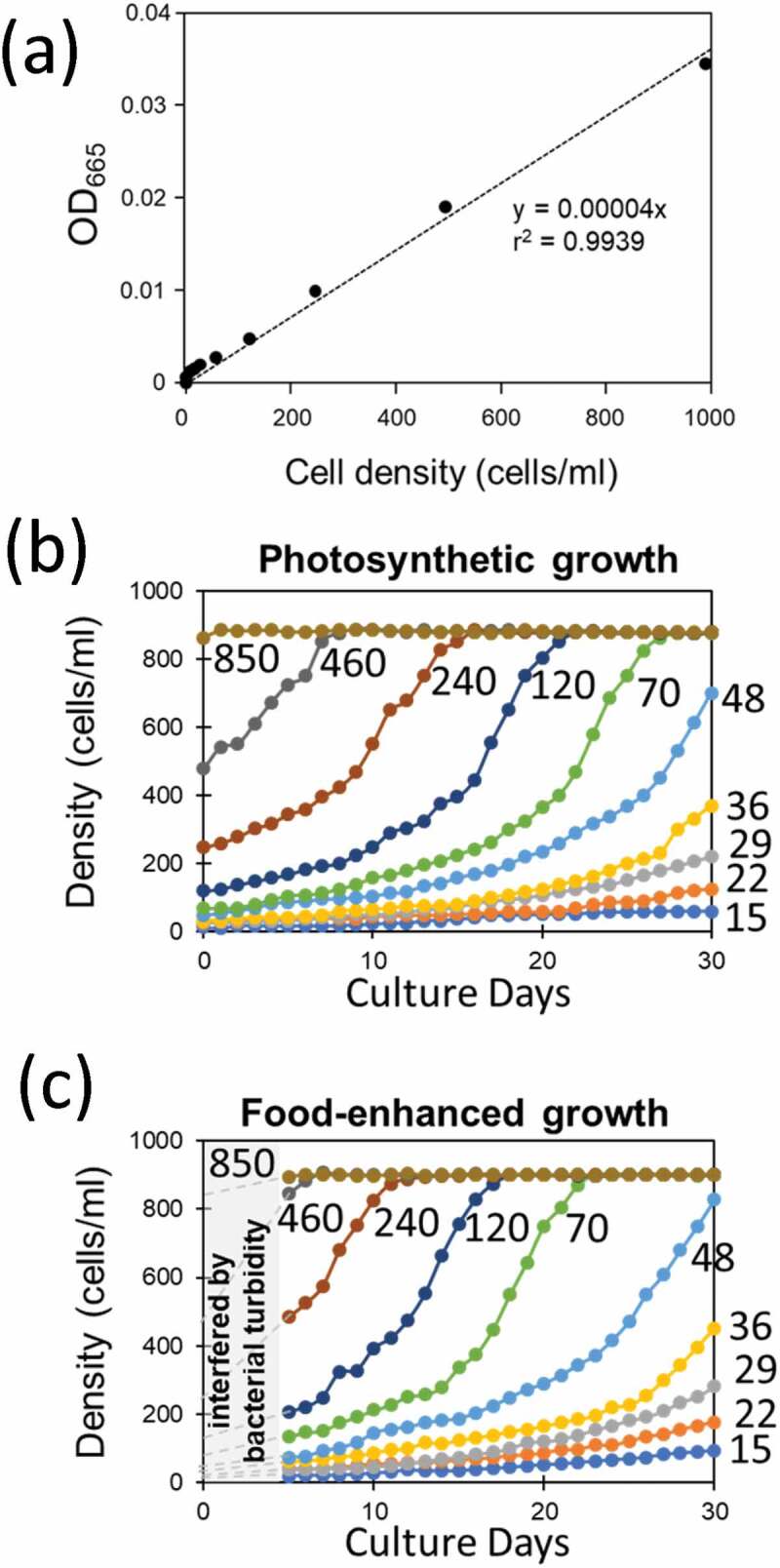
Optically monitored growth of green paramecia. (a) Relationship between OD_665_ and cell density. (b) Effect of initial cell density on the growth of cells under photosynthesis. (c) Effect of initial cell density and addition of bacterized medium on the growth of cells. Optical reading was interfered by the turbidity of food bacteria during initial 5 days of culture. Numbers indicate the initial cell density (cells/ml).

Using a UV-VIS spectrophotometer (UV-1800, Shimadzu Corp., Japan), cellular density of green paramecia was optically monitored at 665 nm (OD_665_) corresponding to the absorbance by chlorophyll *a* (at Q band), after preliminarily obtaining a linear relationship between the microscopically counted cell density and OD_665_ (linearity was found up to 1000 cells/ml; Figure 1(a)).

Growth of green paramecia was optically monitored with OD_665_ (Figure 1(b,c)). As expected initial cell density largely affected the growth of cells propagated solely under photoautotrophic condition (Figure 1(b)) and under photoheterotrophic condition fed with food bacteria (Figure 1(c)). Similar growth patterns were obtained as the growth of green paramecia was optically monitored at different wavelengths, namely at 423 nm (OD_423_) and 520 nm (OD_520_) corresponding to absorption by chlorophylls (Soret band) and chlorophyll-independent turbidity, respectively (data not shown).

**Figure 2.**
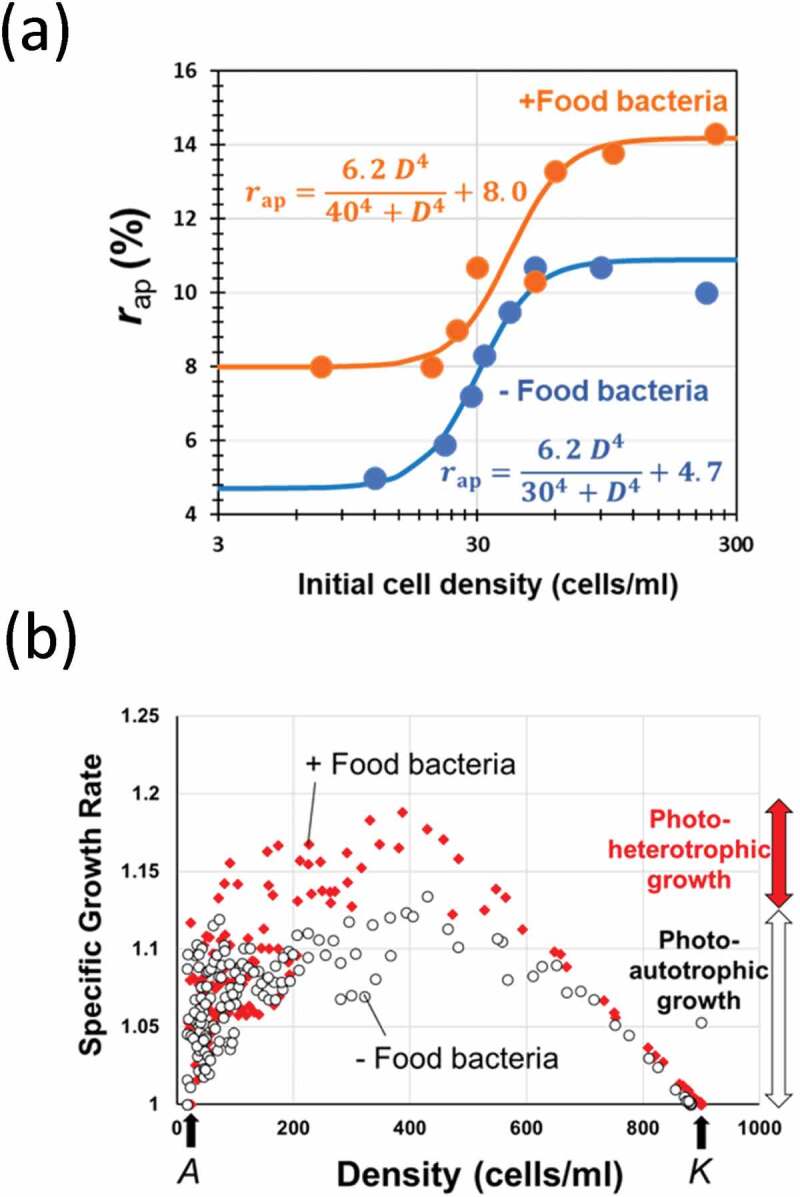
Effect of cell density on the growth pattern in green paramecia in the presence and absence of food bacteria. (a) Relationship between the initial cell density and the apparent growth rate (*r*_ap_). (b) Effect of cell density on the specific growth rate. *A* and *K* values were commonly determined in the presence and absence of food bacteria.

Since the growth of *Paramecium* species could be simulated with logistic equation, we have previously developed an experimental procedure for determination of the ecological factor-sensitive *K* and *r* values which are critical for simulating the growth under a given aquatic condition, by exposing the *Paramecium* samples to the changing environmental stimuli after packing in a semipermeable capsule at high and low densities [29]. Then, the apparent rate of increase (*r*_ap_) allowed in a given environment can be determined with model experiments. Using the data, namely, time (days) required for attaining the 90% confluent, or density of cells at the end of 1 month-long culture, the value for *r*_ap_ in each case starting from varying initial cell density was analyzed (Figure 2(a)).

By starting the culture with *N*_0_ at low initial density of microbial cells, we will observe the increase in population (*N_j_*) on the *j*^th^ day of culture, thus, *r*_ap_ can be expressed as follows:

(8) $$r_{ap}\left(\%\right)=\left(\sqrt[{j}]{\frac{N_{j}}{N_{0}}}-1\right)*100$$

Data suggested that the size of *r*_ap_ largely depends on the initial density (*D*) of culture in both photo-autotrophic and photo-hetrotrophic conditions. By considering the S-shaped increases in *r*_ap_ along with the increase in *D*, the changes in *r*_ap_ were expressed as Hill-type functions of *D* (9; Figure 2(a)). As derivatives of Hill equation are suitable for fitting the sigmoidal curves and they are amazingly applicable to a vast range of kinetics as discussed elsewhere [5], curve-fitting was performed by practically arranging the Hill-type equation (9) assisted by curve fitting with graphical elucidation of Gauss-Newton algorithm (GEGNA) as previously described [5]. By definition, GEGNA iteratively finds the values of the variables which minimize the residual sum of squares (RSS) as often applied to the determination of variables for various kinetic models [30].

(9) $$r_{ap}\left(\%\right)=\frac{r_{max}D^{\alpha }}{K_{D}^{\alpha }+D^{\alpha }}+r_{0}$$

Figure 2(b) shows the effect of changing cell density on the coming growth pattern resulting in altered specific growth rate (equivalent to the growth per capita in higher organisms) in green paramecia in the presence and absence of food bacteria. By plotting the data in this way, the presence of upper and lower population limit determined as *K* and *A* values could be visualized (Figure 2(b)). Interestingly, green paramecia both under photo-autotrophic and photo-heterotrophic conditions commonly showed the presence of *A* and *K* values which were almost identical under two distinct trophic conditions, suggesting that the upper and lower thresholds for density effects are independent from the trophic modes.

The above analyses (Figures 1 and 2) implied that logistic models can be applied as predicted by the presence of density-dependently altered growth rate (*r*_ap_) and upper density limit (*K*). In addition, there could be Allee’s density effect although the elucidated *A* value is hardly resolved from zero.

### Simulation of green paramecium growth using the novel Allee model

**Figure 3.**
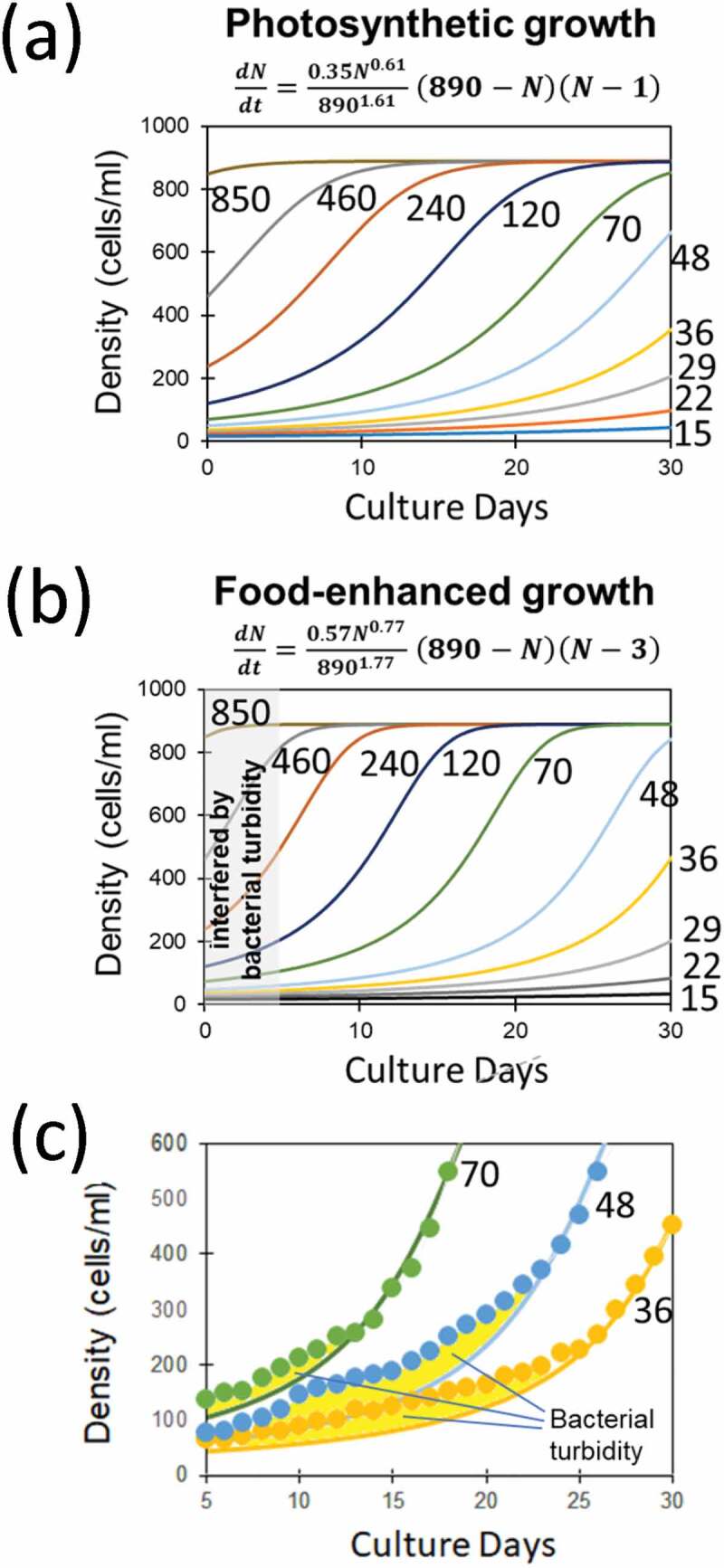
Growth of green paramecia simulated with the novel Allee model. The growth curves simulated with various initial cell densities solely under photosynthesis (a) and with food bacteria (b). (c) Estimation of the uptake of food bacterium by green paramecia shown as the gaps (filled with yellow color) between optically monitored data points (dots) and simulated growth curves. Novel mathematic model for flexible density effect with Allee threshold (7) was used for Gauss-Newton algorithm-based curve fitting. Numbers indicate the initial cell density (cells/ml).

The novel equation (7) for logistic model with flexible Allee effect was used for regeneration of growth curves for green paramecia through simulative manner. Simulated growth curves starting from various initial cell densities solely under photosynthesis (photo-autotrophic condition) were performed after curve-fitting through GEGNA, thus employing following parameters, namely, *α *= 0.61, *r* = 0.35, *K* = 890, and *A* = 1 (Figure 3(a)). Similarly, simulation of growth under combination of photosynthesis and food bacteria (photo-heterotrophic condition) was performed by employing following parameters: *α *= 0.77, *r* = 0.57, *K* = 890, and *A* = 3 (Figure 3(b)). There were apparent gaps between the recorded cell density data points (calibrated after OD_665_) and simulated curves (Figure 3(c)). There is tendency that such gaps due to unexpected signals last for longer period as the initial *Paramecium* density is lowered, consistently supporting the view that the difference must be due to the turbidity attributed to the initially added food bacteria which is apparently acting for over-estimating the *Paramecium* cell density, and the efficiency for digesting out the bacterial population depends on the *Paramecium* cellular density.

It is conclusive that growth of photo-autotrophically and photo-heterotrophically propagated green paramecia was shown to be regulated by flexible density effect.

### Single mutation revealed the hidden Allee effect in cyanobacteria

The mathematical model applied for simulating the growth of green paramecia under density effect was also used for growth simulation of cyanobacterial cells (*Synechocystis* sp. PCC6803 strain). Here, the cells of wild type and Δ*pxcA* mutant line of PCC6803 were grown in BG11 medium supplemented with 20 mM HEPES (pH 8.0) at 30 °C with continuous illumination at 50 µmol photons m^−2^ s^−2^ for analysis of growth rates.

**Figure 4.**
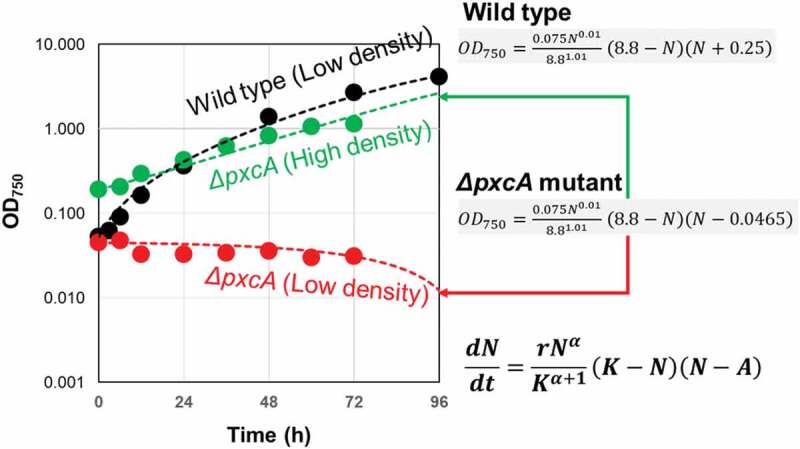
Allee effect found in Δ*pxcA* mutant cell line of cyanobacteria (*Synechocystis* spp. PCC6803). Optically monitored growth (OD_750_) of wild type and Δ*pxcA* mutant lines were compared. For each cell line, novel mathematic model for flexible density effect with Allee threshold (7) was used for Gauss-Newton algorithm-based curve fitting.

Under standard photo-autotrophic culturing condition, growth of wild type cells and Δ*pxcA* mutant cells was compared. At low inoculation densities (between 0.045 and 0.053 OD_750_), Δ*pxcA* mutant cell line failed to reproduce while wild type cells rapidly grew to the logarithmic range (Figure 4). On the other hand, Δ*pxcA* mutant cells successfully multiplied as the inoculation density was elevated by fourfold (up to 0.191 OD_750_), suggesting the Δ*pxcA* mutant cells are clearly under Allee-type density effect.

By employing the mathematical model (7) used for simulation of green paramecium growth, curve fitting was performed through GEGNA, to regenerate the growth curves for wild type and Δ*pxcA* mutant (Figure 4). Followings are the parameters used for simulating the growth of Δ*pxcA; α *= 0.01, *r* = 0.075, *K* = 8.8, and *A* = 0.047 (Figure 3(a)). With this model, only the changes in initial cell density drastically alter the fate of culture, i.e., growth is allowed above *A* value (0.047) and no growth occurs below or around *A*. Note that the parameters used for simulating the growth of wild type cells were almost identical except for *A* = −0.24, which is negative value thus, the cells are completely free from suppression at low density. These results indicate that cyanobacteria has overcome the harm from low-density effect in the course of evolution upon acquisition of photosynthesis-related *pxcA* gene.

This is the first observation that single-gene mutation in photosynthetic organism results in the loss of function in the propagation pattern at low density, possibly under Allee effect.

### Photosynthetic kinetics behind the upper growth limit

The density effect determining the upper limit of photosynthetic growth around *K* can be partially explained by the limitation of collective harvesting of light energy. It is trivial that net photosynthesis (*P_net_*) is a consequence of gross photosynthetic activity (*P*, represented by O_2_ evolution or CO_2_ uptake) and respiration (*R*), thus, the *P_net_* can be expressed as follows:

(10) $$P_{net}=P-R$$

Platt and Jasby [31] have proposed that the modified Michaelis-Menten equation effectively reproduces the relationship between the light intensity and photosynthesis of marine algae, by re-formalizing the velocity of photosynthesis (*P*) as the function of given light intensity (*J*), where *P*_max_ is maximal gross photosynthetic velocity under ideally high intensity of light, and *K*_j_ is equivalent to the Michaelis constant for the light intensity to achieve 1/2 *P*_max_.

(11) $$P=\frac{P_{max}J}{K_{j}+J}$$

We have previously confirmed that photosynthetic activities in PCC6803 cells under simulated solar light and artificial white light were shown to obey the Platt-Jasby model [32,33]. As discussed earlier [34,35], higher generality can be conferred to the mathematical model by converting the Michaelis-Menten-type equation (11) into a Hill-type equation (12).

(12) $$P=\frac{P_{max}J^{\alpha }}{K_{j}^{\alpha }+J^{\alpha }}$$

As classically pointed by many researchers [36], transmittance (*T*) of light through the layers of green cells can be expressed by Beer-Lambert law as follows:

(13) $$T=e^{-ax}$$

where *a* and *x* are absorption coefficient and the depth of culture (or density of cells), respectively. In case of terrestrial plant canopy (leaf layers), we have previously made estimation on the sum of light available for photosynthesis within the canopy structure in the form of convergent geometric progression [35]. Similarly we can summarize the relationship between algal and cyanobacterial densities and collective capturing of incident light as follows:

(14) $$\overset{\infty }{\underset{i=0}{\sum }}JT^{i}=\underset{k\to \infty }{\lim}\overset{k}{\underset{i=0}{\sum }}JT^{i}=\frac{J}{1-T}$$

where *i* is number of photosynthetic cells across the path of light at a given cellular density, *J* is intensity of incident light, and *T* is transmittance by algal/cyanobacterial layer of cells. Above equation suggests that light is collectively filtered by green cells, and the yield of photosynthetically active light convergently attains the maximal level. By introducing this term in place of the term for light intensity in Platt-Jasby equation (11), following equation can be derived:

(15) $$\overset{k}{\underset{n=1}{\sum }}P_{n}=\frac{P_{max}\frac{J}{1-T}}{K_{j}+\frac{J}{1-T}}$$

where *P_n_* denotes the photosynthesis in the *n*^th^ degree of increased green cell density. Since the collective light yield rapidly converges, *k* can be replaced with ∞∞ in a practical sense. Therefore, this relationship can be rearranged to conclude that Michaelis constant can be practically altered. To widen the application range, this relationship can be also expressed as Hill-type equation (16).

(16) $$\overset{k}{\underset{n=1}{\sum }}P_{n}=\frac{P_{max}J^{\alpha }}{{\left\{K_{j}\left(1-T\right)\right\}}^{\alpha }+J^{\alpha }}$$

The above mathematical model clearly suggests that there should be converging level of total photosynthesis along with the increase in cell density. However, to maintain the surplus in photosynthesis (related to growth rate) at maximal level, the cell density-dependent increase in respiration must be minimized as described in equation (10). By this way, maximal cell density attained can be naturally determined.

### Discussion on the possible cell-cell communication among photosynthetic organisms

In the cells derived from higher organisms such as human cells under tissue culture, tight and integrated networks of chemical communication among self- and non-self cells govern the mode of proliferation at cellular level. For example, successful cell proliferation during tissue culturing of human and animal cells often requires relatively high initial cell density since the cells are the source of autocrine-derived growth factors [37,38]. In such cases, certain population size may contribute to the priming of extracellular microenvironment. In this point, the minimal cellular density required for allowing the proliferation of the cells could be considered equivalent to Allee threshold.

In the case of green paramecium culture (Figure 2) and *pxcA* mutant in PCC6803 cells (Figure 4), we have practically determined the Allee thresholds. However, the mechanism how these photosynthetic cells are sensing its population size is still unknown. One of likely mechanisms to be discussed could be alleviation of light stress by certain size of pigmented population. This point should be clarified in the future works. Or, by analogy to microbial quorum sensing mechanisms [2,3], the mode of chemical communications among photosynthetic organisms could be elucidated.

## Conclusion

In the upper half of the present perspective work, we have rearranged a novel equation for highly flexible density effect model combined with Allee threshold. In the lower half of this article, we reported an Allee-like density effect in green paramecia and cyanobacteria despite Allee effect has been rarely observed in microorganisms to date (except for few heterotrophic ciliate species and genetically engineered bacteria).

Optically monitored growth of green paramecia was shown to be obeying the Allee-like weak density effect under photosynthetic conditions with and without food bacterium supplementation.

Wild type PCC6803 cells were shown to be insensitive to such low-density condition as consistent with our empirical knowledge often observing the growth of organisms after severe dilution (data not shown). By showing a good contrast, a mutant line of PCC6803 impaired with a photosynthesis-related *pxcA* gene (involved in CO_2_ transport) was determined to be highly sensitive to low-density condition. The mutant cells failed to propagate at low cellular density while the cells started logarithmic growth at fourfold higher inoculating density. This is the first observation that single-gene mutation in an autotrophic organism alters the sensitivity to Allee effect.

These results are supporting our view that that photosynthetic bacteria, chiefly cyanobacteria, have overcome the negative impact by low-density effect in the course of evolution through acquisition of photosynthesis-associated genes such as *pxcA*.
